# Glutathione is an aging-related metabolic signature in the mouse kidney

**DOI:** 10.18632/aging.203509

**Published:** 2021-09-07

**Authors:** Eunyong Ahn, Jueun Lee, Jisu Han, Seung-Min Lee, Ki-Sun Kwon, Geum-Sook Hwang

**Affiliations:** 1 Integrated Metabolomics Research Group, Western Seoul Center, Korea Basic Science Institute, Seodaemun-Gu, Seoul 03759, Korea; 2 Aging Research Center, Korea Research Institute of Bioscience and Biotechnology, Yuseong-Gu, Daejeon 34141, Korea; 3 Aventi Inc., Yuseong-Gu, Daejeon 34141, Korea; 4 Department of Chemistry and Nano Science, Ewha Womans University, Seodaemun-Gu, Seoul 03760, Korea

**Keywords:** metabolomics, transcriptomics, renal aging, glutathione metabolism

## Abstract

The ability to maintain systemic metabolic homeostasis through various mechanisms represents a crucial strength of kidneys in the study of metabolic syndrome or aging. Moreover, age-associated kidney failure has been widely accepted. However, efforts to demonstrate aging-dependent renal metabolic rewiring have been limited.

In the present study, we investigated aging-related renal metabolic determinants by integrating metabolomic and transcriptomic data sets from kidneys of young (3 months, *n* = 7 and 3 for respectively) and old (24 months, *n* = 8 and 3 for respectively) naive C57BL/6 male mice. Metabolite profiling analysis was conducted, followed by data processing via network and pathway analyses, to identify differential metabolites. In the aged group, the levels of glutathione and oxidized glutathione were significantly increased, but the levels of gamma-glutamyl amino acids, amino acids combined with the gamma-glutamyl moiety from glutathione by membrane transpeptidases, and circulating glutathione levels were decreased. In transcriptomic analysis, differential expression of metabolic enzymes is consistent with the hypothesis of aging-dependent rewiring in renal glutathione metabolism; pathway and network analyses further revealed the increased expression of immune-related genes in the aged group.

Collectively, our integrative analysis results revealed that defective renal glutathione metabolism is a signature of renal aging. Therefore, we hypothesize that restraining renal glutathione metabolism might alleviate or delay age-associated renal metabolic deterioration, and aberrant activation of the renal immune system.

## INTRODUCTION

Aging accelerates multisystemic deterioration and increases the risk of developing metabolic diseases or syndromes. The ability to modulate systemic metabolite levels through various mechanisms, such as tubular secretion, glomerular filtration, and catabolism, represents a crucial strength of kidneys in the study of metabolic syndrome and aging [1]. Among aging-related unfavorable dysregulations in various organs, renal aging is considered a highly complex interplay of genetic, epigenetic, and environmental changes; moreover, it is mutually correlated with the incidence of systemic pathophysiological changes [2]. The characteristics of aged kidneys include structural changes and subsequent functional changes, such as a declining glomerular filtration rate (GFR) and tubular dysfunction [2]. Although the mechanisms underlying progressive renal injury in chronic kidney diseases (CKDs) differ from the gradual changes observed during the renal aging process, renal aging decreases the kidney resilience, and the prevalence of CKD increases with age [3, 4].

Due to structural and functional changes, renal aging contributes to alterations in related biological processes. In aged kidneys, signaling pathways are rewired, and unhealthy mitochondria lead to the increased accumulation of oxidative stress [5]. It is widely accepted that oxidative stress contributes to tissue damage, thereby leading to pathological changes during the aging process. In both murine and human studies, researchers reported that aging was associated with increases in reactive oxygen species (ROS) generation and alterations in ROS removal ability [6]. Furthermore, renal oxidative stress is considered a major factor underlying the initiation of diabetic nephropathy [7]. Thus, an adequate glutathione (GSH) supply in the kidneys is critical for maintaining renal function [8]. The protein expression of renal Klotho, a transmembrane protein modulating diverse aging-associated pathways, is known to be decreased in aged mice compared to young mice [9]. Decreasing Koltho levels further impact the suppression of FGF and the alteration of Wnt signaling pathways [10]. Although whether leukocyte-derived inflammatory activation is the cause or effect of aging remains unclear, increased glomerular macrophage infiltration was histologically observed in healthy aged mice [2, 11].

Despite the significant role of kidneys in aging-associated systemic metabolic deterioration, efforts to comprehensively demonstrate this mechanism through omics-level profiling analysis have been limited in the study of renal aging. Thus, we herein investigated aging-related renal metabolic determinants through the integration of metabolomic and transcriptomic data sets of mouse kidneys of different ages. We performed aqueous metabolite profiling analysis of 15 mouse kidney samples, and the acquired metabolomics data were processed via network and pathway analyses to identify differential metabolites. Transcriptomic analysis was also performed on 6 mouse kidney samples, revealing altered expression levels of residing metabolic enzymes related to our metabolic profiling analysis.

## RESULTS

### Profiling of aqueous metabolites revealed differential glutathione levels in the kidneys of young and old mice

**Figure 1:**
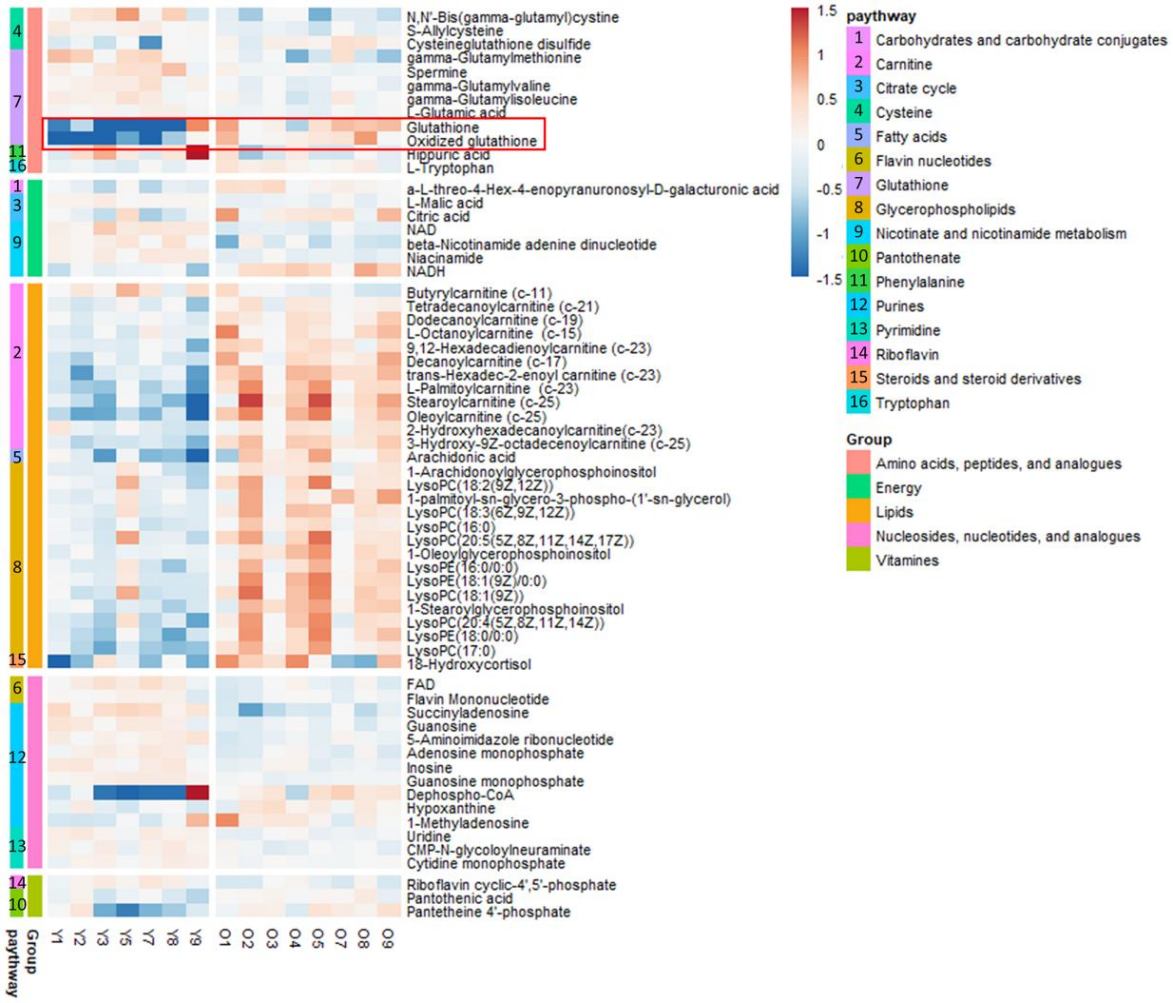
**Heat map of metabolites in the kidney tissues of young (*n* = 7) and old (*n* = 8) mice *in vivo*.** The metabolites were plotted and further curated according to their residing KEGG pathways. The heat map was color-coded according to the log 2 transformed fold change in the measured relative intensities of each sample.

To explore the aging-dependent metabolic signature, we performed LC-MS-based nontargeted aqueous metabolic profiling analysis followed by *in silico* identification. Mouse kidney tissue samples were obtained from young (*n* = 7) and old (*n* = 8) mice. PCA and PLS-DA analysis revealed strong separation between two groups based on spectral data obtained in positive and negative modes. Since QC samples clustered as a group, we confirmed the reproducibility of our LC-MS-based analytical platform (Supplementary Figure 1). Q2 discrimination rates of 84.2% and 86.2% were obtained for the spectral data obtained in the positive and negative modes, respectively (Supplementary Figure 1). The heatmap illustrates the levels of all the identified metabolites and the pathway-specific patterns (Figure 1). While GSH and oxidized GSH were higher by 2.37- and 2.98-fold, respectively, in the old group compared with the counterpart, GSH-related metabolites such as gamma-glutamyl dipeptides were lower in the old group compared with the young group (Supplementary Table 1). Lipids such as acyl-carnitines, Lyso phosphatidylcholines (PCs) and Lyso phosphatidylethanolamines (PEs) were higher in the kidney tissues of old mice compared to their counterparts. Interestingly, our transcriptomic data showed that a known lipid synthesis driver of skeletal muscle, Acsl6 [12], was significantly overexpressed in the old group compared to the young group. Two other Acsl isotypes that mainly play roles in energy expenditure and lipid oxidation, Acsl1 and Acsl5, were not different (Supplementary Figure 2). Most nucleosides, nucleotides and their analogs were slightly lower in the old group compared to the young group. Intriguingly, we observed higher levels of CoA synthesis metabolism intermediates, such as dephospho-CoA, pantothenic acid and pantotheine 4-phosphate, in old mouse kidneys compared to their counterparts.

### Network analysis revealed significant alterations in kidney glutathione levels

**Figure 2:**
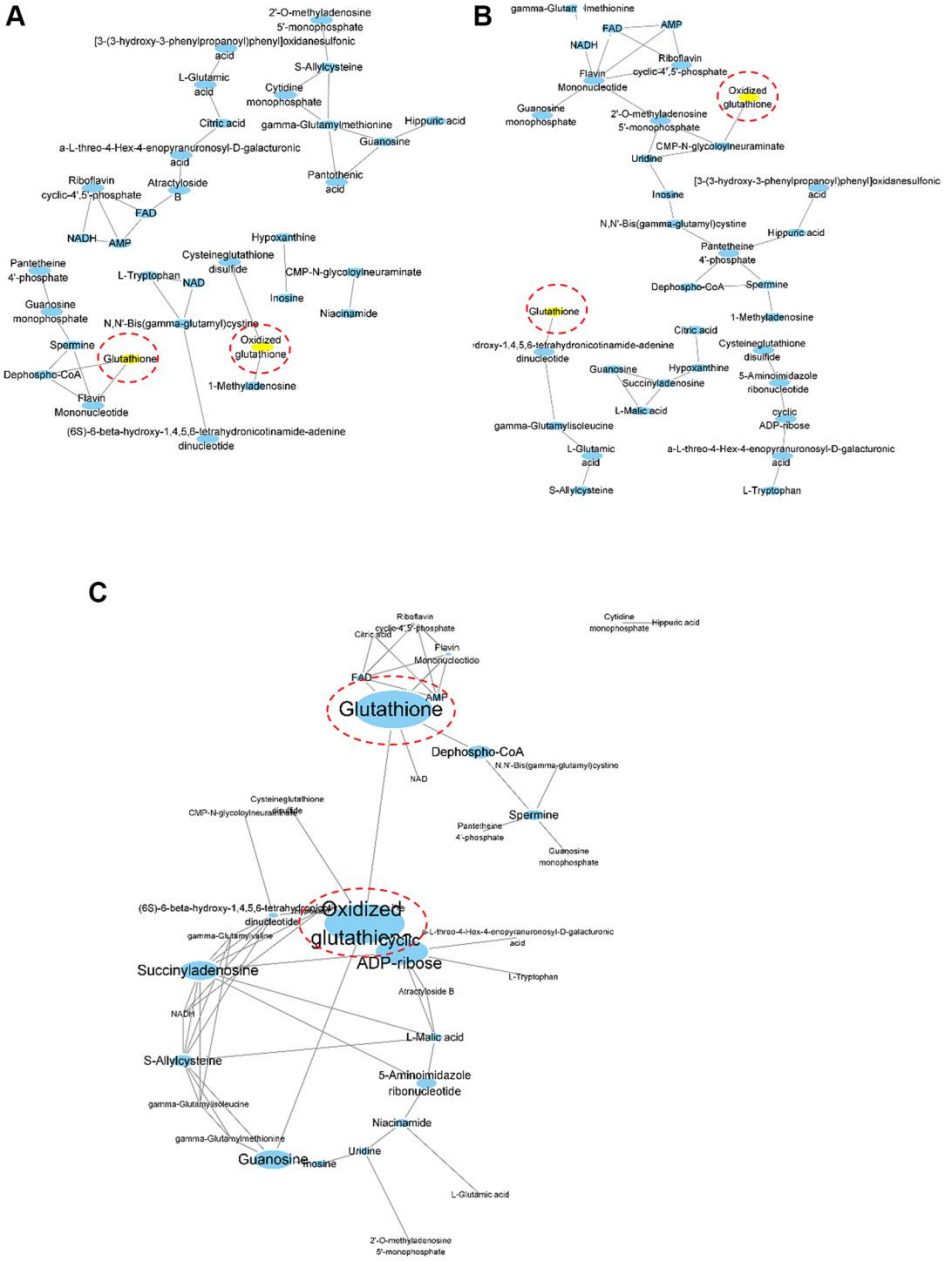
**Correlation coefficient-based network analysis results.** Network visualization of the correlation-based relationships among profiled aqueous metabolites was performed for young (**A**, *n* = 7), old (**B**, *n* = 8) and mixed (**C**, *n* = 15) groups. Oxidized glutathione and glutathione are highlighted in yellow for (**A)** and (**B**). The width and height of the nodes were scaled using the stress centrality measurements for (**C**).

To identify the metabolic determinants in aged mouse kidney tissue, we performed correlation coefficient-based network analysis (Figure 2). The relative abundances of aqueous metabolites from the young (Figure 2A), old (Figure 2B), and mixed groups (Figure 2C) are represented as nodes in the network graph. Here, only the metabolites with HMDB identifiers were included in the analysis. The presence of edges, whether two nodes are connected in a network, is often determined based on the correlation coefficient of two variables. In our research, the presence of edges was determined according to the resampling strategy-based PCLR algorithm [13] followed by the differential network analysis. Interestingly, GSH and oxidized GSH were differentially associated with other metabolites between the young and old groups as determined by network differential connectivity analysis (Table 1). In the network built from the metabolomics data obtained from young mouse kidney tissue, GSH and oxidized GSH were associated in a more complex manner (Figure 2A), whereas they were unassociated in the network built by the metabolomics data from old mouse kidney tissue (Figure 2B). Furthermore, we built a correlation-based network using both groups to elucidate the relationship between aging-dependent changes in metabolites. To address the key metabolites in the results, we calculated the stress measurement, which is the number of passing shortest pathways and a proxy of the importance in the network [14]. Based on the stress measurements, GSH and oxidized GSH were again determined to be differentially connected (Figure 2C). Therefore, our concordant results from the network differential connectivity and stress measurement analyses demonstrated that GSH and oxidized GSH are key differential metabolites and simultaneously representative features in a mouse aging kidney model.

**Table 1:** Network differential connectivity analysis.

**Metabolite**	**pval_corr**	**pval_mi**
Glutathione	0.06	0.02
1-Methyladenosine	0.14	0.04
Hippuric acid	0.02	0.06
Dephospho-CoA	0.02	0.06
Atractyloside B	0.04	0.16
Oxidized glutathione	0.04	0.24
Pantothenic acid	0.04	0.36
L-Malic acid	0.04	0.56
2′-O-methylaenosine monophosphate	0.02	0.86

Network differential connectivity analysis results of two networks built by the metabolomic data from young (A, *n* = 7) and old (B, *n* = 8) mouse kidney tissues. *P*-values were calculated based on the permutation in the algorithm. Correlation and mutual information were implemented for the analysis as previously described [47], and the *p*-values from each method using either correlation coefficient or mutual information referred to as pval_corr and pval_mi. Only the metabolites with *p*-values below 0.05 determined via any method are shown above.

### Differences in riboflavin, nicotinamide, and purine metabolism in aged mouse kidneys identified by differential metabolic pathway analysis

**Figure 3:**
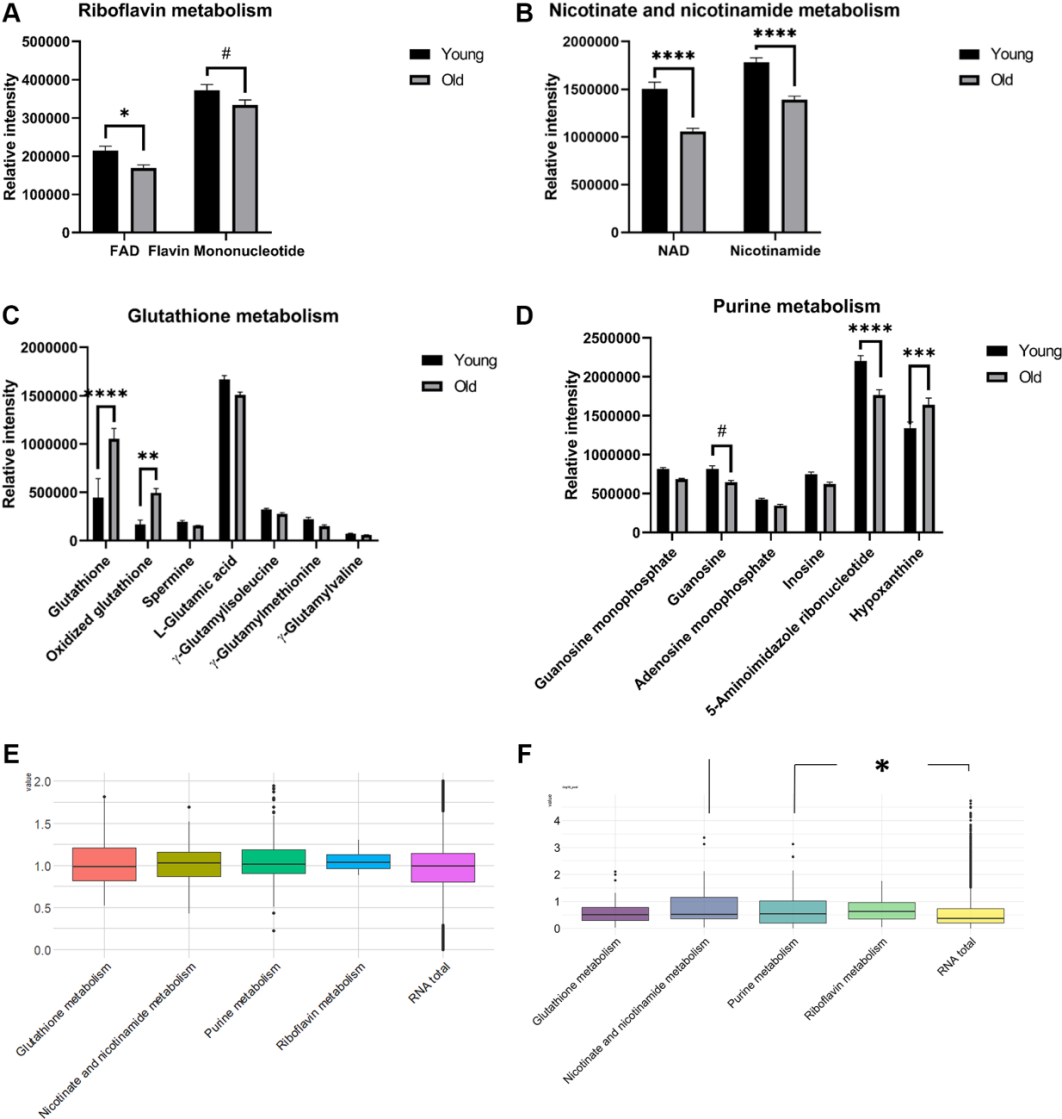
**Barplots of the relative intensities of metabolites in differential pathways in aged mouse kidney tissues (A**–**D, *n* = 7 and 8 for young and old groups, respectively).** The means and SEMs of the relative intensities determined by LC-MS are plotted. *P*-values were calculated by two-way ANOVA with Sidak’s multiple comparison tests to see intergroup differences for (**A**–**D**). Boxplots of the log2 transformed fold changes of genes annotated in differential pathways (**E**, *n* = 3 for both the young and old groups). Boxplots of the –log_10_(*P*-values) determined by *t*-tests to assess the differential expression of genes between the old and young groups (**F**, *n* = 3 for both the young and old groups). The distributions of –log_10_ *P* values from each pathway were tested and compared to those in the whole transcriptome by Wilcoxon rank-sum tests. (*p*-values: ^#^< 0.1, ^*^< 0.05, ^**^< 0.01, ^***^< 0.001, ^****^< 0.0001).

To further understand the differential metabolism in aged mouse kidney tissue, pathway analysis of metabolomics profiling data was performed. False discovery rate (FDR) values from 9 metabolic pathways were less than 0.05; simultaneously, at least two residing metabolites were identified from profiling analysis (Supplementary Table 2). Among these selected pathways, 4 metabolic pathways (riboflavin, nicotinate and nicotinamide, GSH, and purine) had impact values greater than 0.2. Flavin adenine dinucleotide (FAD) (*p*-value = 0.0237) in riboflavin metabolism (Figure 3A) and nicotinamide adenosine dinucleotide (NAD) (*p*-value < 0.0001) and nicotinamide (*p*-value < 0.0001) in nicotinate and nicotinamide metabolism (Figure 3B) were significantly lower in the old group compared to their counterparts. Hypoxanthine (*p*-value = 0.0002) in purine metabolism was higher in the old group compared to its counterpart, whereas 5-aminoimidazol (*p*-value = 0.0001) was significantly lower in the old mice compared to the young mice (Figure 3D). Intriguingly, GSH (*p*-value < 0.0001) and oxidized GSH (*p*-value = 0.0027) were significantly higher in the old group (Figure 3C), and GSH metabolism was selected as a differential aging-dependent pathways; however, γ-glutamyl amino acids without HMDB identifiers were not included in the current pathway analysis.

To determine whether the metabolite levels potentially differ due to transcriptomic alteration, transcriptomic data for genes annotated within these four pathways were used for the descriptive analysis based on the KEGG database. For each pathway, 8 (riboflavin), 40 (nicotinate and nicotinamide), 53 (GSH), and 135 (purine) genes were annotated. To answer if the expression levels of genes residing in certain pathways are specifically up or down-regulated by aging, we compared the distributions of log2 fold changes in RNA expression by aging between each pathway and the whole transcriptome (Figure 3E). Additionally, since the metabolic pathway could be rewired by significant and non-monotonous changes of genes, we also explored if these four pathways are more rewired compared to the whole transcriptome. To this end, the distribution of the *p*-values from the *t*-test between young and old groups was presented (Figure 3F). Although the fold change distributions were not monotonously skewed toward one direction, the *p*-values from nicotinate and nicotinamide metabolism and purine metabolism were significantly smaller than those from the system-level transcriptome (*p*-values: 0.0113 and 0.0171, respectively). Notably, the *p*-value estimate from the Wilcoxon rank-sum test obtained by comparing the *p*-value distribution of genes in the GSH pathway from the *t*-test to that in the whole transcriptome was 0.0648; however, the estimate was not statistically significant at the 0.05 level.

In accordance with our observed lower levels of nicotinamide metabolism intermediates in aged mice, previous studies revealed that the NAD+ and NAMPT levels were decreased in multiple organs during aging [15]. In mice with aging-induced diabetes, nicotinamide mononucleotide (NMN), a key NAD+ intermediate, also ameliorated glucose intolerance via the restoration of NAD+ levels [16].

### Rewiring of glutathione metabolism in aged mouse kidneys

**Figure 4:**
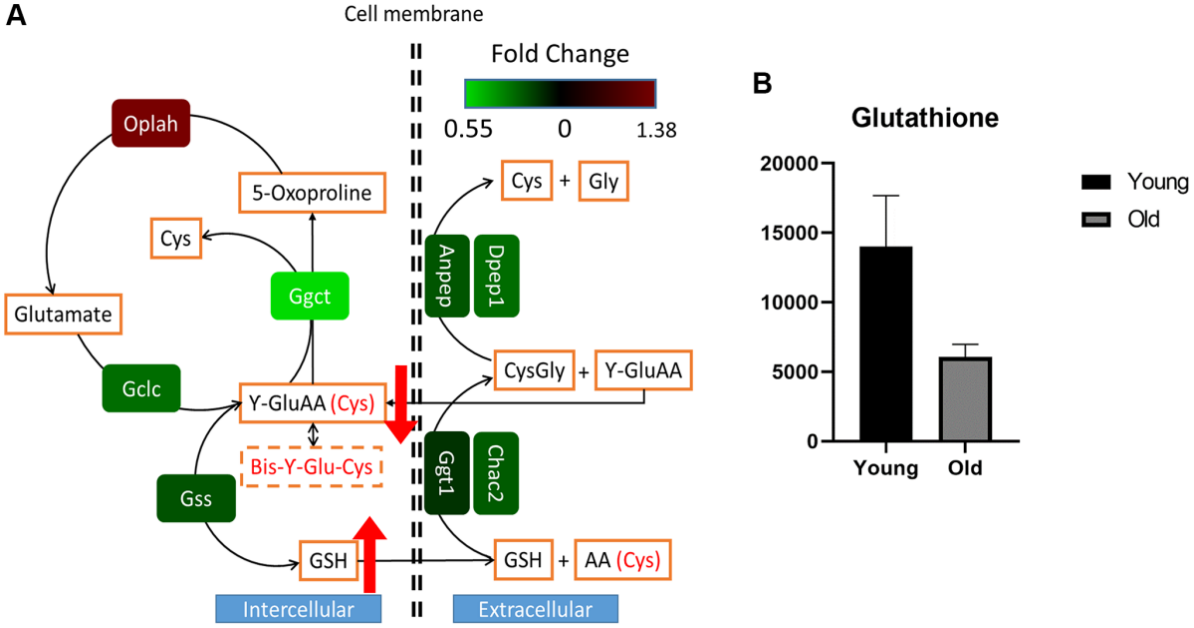
**Rewired glutathione metabolism in aged mouse kidney tissue.** The transcriptomic profile was plotted according to the glutathione metabolic pathway (**A**). Relative intensities of the serum glutathione levels in young (*n* = 4) and old (*n* = 4) mice as determined by LC-MS analysis (**B**). Circulating systemic serum glutathione appeared to be decreased in the old group compared to the young group, but the difference was not significance (*p*-value: 0.1143). Wilcoxon rank sum test was performed to determine whether glutathione was decreased in the old group.

To further elucidate the aging-related differences of GSH metabolism in our mouse kidney experiments, we comprehensively explored GSH metabolism via transcriptomic data. The expression levels of residing metabolic genes were significantly varied as follows. Gamma-glutamyl cyclotransferase (Ggct), GSH synthetase (Gss), and cation transport regulator 2 (Chac2) were expressed at lower levels in the old group than in the young group (adjusted *p*-values: <0.0001, 0.0609, and 0.0957, respectively), and 5-oxoprolinase (Oplah) was expressed at higher levels in the old group (adjusted *p*-value: 0.0407) than in the young group (Figure 4A, Supplementary Table 3). While GSH and oxidized GSH levels were significantly enriched in the old group, the gamma-glutamyl amino acid levels were slightly decreased in the old group (Supplementary Figure 3, Supplementary Table 1). Our results did not reveal an obvious relationship or causality of the phenomenon, potentially due to the lower levels of gamma-glutamyl transferases (GGTs) or GSH-specific gamma-glutamyl cyclotransferase 2 (CHAC2) enzymes, which catalyze GSH catabolism and gamma-glutamyl amino acid synthesis. In fact, concordant with our results, increases in GSH and oxidized GSH levels and decreases in gamma-glutamyl amino acid levels attributed to rewiring at the transcription levels of related genes were reported in malignant human kidney tumors [17, 18]. Furthermore, we simultaneously observed decreased GSH levels in serum samples from the old group (Figure 4B). Considering that the kidneys regulate GSH catabolism and systemic cysteine homeostasis, our results cautiously suggest that the systemic decrease in circulating GSH levels is attributed to altered GSH catabolism and increased GSH and oxidized GSH pool sizes in aged mouse kidneys.

### Transcriptomic analysis of differential metabolic pathways in aged mouse kidneys

In addition, revealing rewired GSH metabolism during renal aging by metabolomic analysis, transcriptomic data were bioinformatically analyzed to identify differential metabolic pathways at the expression level. KEGG pathway analysis using transcriptomic data revealed that immune-related pathways, such as the intestinal immune network for IgA production, chemokine signaling, natural killer cell-mediated cytotoxicity, Jak-STAT signaling, phagosomes, T cell receptor signaling, complement and coagulation cascades, antigen processing and presentation, and Toll-like receptor signaling pathways, were enriched in the old group compared to the young group (Supplementary Figure 4A). A previous study also revealed that genes in immune response pathways were most differentially expressed at the RNA level in aged mouse glomerular podocytes [19]. In fact, published data support that the immune system plays an underlying role in renal aging; reduced renal fibrosis and cellular senescence were observed in aged animals after bone marrow transplants from young mice [2, 20]. In contrast, pathways related to cellular protein synthesis, such as the mRNA surveillance, spliceosome, and protein processing in endoplasmic reticulum pathways, were downregulated in the old group compared to their counterparts. Two cytochrome P450 enzymes mediated pathways, the retinol and drug metabolism pathways, were upregulated in the old group. Members of tight junction and oxidative phosphorylation pathways were also expressed at lower levels in the old group.

To understand a potential association between gene sets in the mouse kidney aging model, we utilized the GScluster gene set clustering method of Kin et al. (Supplementary Figure 4B) [21]. In this method, PPI and KEGG databases for *Mus musculus* were used to determine possible interactions among groups of genes. Two clusters were identified as follows. Gene sets in KEGG pathways related to the immune system, natural killer cell-mediated cytotoxicity, B cell receptor signaling, *T* cell receptor signaling, and FC epsilon RI signaling pathways were clustered into one group. In the other cluster, metabolic pathways involving cytochrome P450 genes, such as xenobiotic metabolism by cytochrome p450, drug metabolism, retinol metabolism and linoleic acid metabolism, were grouped together.

Gene Ontology analysis was performed to explore the enriched gene ontologies in the mouse kidney aging model. The top 10 gene ontology terms from the analysis were plotted for each of 3 categories: cellular component (CC), biological process (BP), and molecular function (MF) (Supplementary Figure 5). Interestingly, most of the top enriched gene ontology terms in the BP category were related to the immune response. GSEA was also performed using our transcriptomic data. A total of 3974 *Mus musculus* gene sets were obtained from mSigDB, and 3352 gene sets were filtered out by gene set size filters (min = 1, max = 500) [22, 23]. The remaining 622 gene sets were used in the analysis. In concordance with other transcriptomic data analysis results, most of the enriched gene sets in the old group were related to the immune response (Supplementary Table 4).

## DISCUSSION

A main characteristic of aging is complex metabolic syndrome; in particular, the renal aging process is mutually correlated with systemic metabolic deterioration. However, although efforts are ongoing to restrain aged kidney metabolism, the factors underlying the causes and effects of age-related renal metabolic dysfunction are largely unknown. To elucidate this comprehensive metabolic alteration, we conducted integrated metabolic and transcriptomic data analysis by utilizing mouse kidney tissues of different ages.

We performed metabolic profiling analysis, revealing aging-dependent metabolic dysregulation. It is well established that changes in lipid metabolism are distinct characteristics of the renal aging process [24]. A previous study has demonstrated that decreased fatty acid oxidization capacity is a main factor underlying age-related lipid imbalance and accumulation [25]. Recently, gene expression analysis of various mammalian tissues, including the liver, kidney, and brain, revealed that the expression levels of genes involved in lipid oxidation and lipid modification were negatively correlated with lifespan [26]. Additionally, the protein and RNA levels of acetyl coenzyme A were reportedly decreased in aged mouse samples compared to their counterparts [27]. In agreement with previous research, we found that the levels of lipid species and CoA synthesis metabolism intermediates in aged kidneys were increased compared with those in young mouse kidneys in our *in vivo* mouse study (Figure 1). Acsl6, an acyl-CoA synthetase that drives acyl coenzyme A toward lipid synthesis in skeletal muscle tissue, was expressed at significantly higher levels in aged kidneys than in young kidneys (Supplementary Figure 2). Although an experimental study has not been conducted to determine whether Acsl6 plays the same role in kidney tissue as it does in skeletal muscle, we did herein simultaneously observe increased Acsl6 gene expression and lipid accumulation in aged mouse kidneys. Our metabolic profiling results also revealed that most intermediates in nucleic acid metabolism were decreased in aged mouse kidneys. The cytosolic ribonucleotide and deoxyribonucleotide metabolism occur in mitochondria, and mitochondrial dysfunction limits the level of nucleotides, thereby resulting in genomic instability. It is well established that aging is associated with a decreased mitochondrial function [28]. Collectively, aging-associated mitochondrial dysfunction could limit and imbalance nucleoside metabolism in aged mouse kidneys.

Furthermore, the aged mouse group showed significantly higher levels of GSH and oxidized GSH but lower levels of gamma-glutamyl amino acids than the young mouse group. Intriguingly, similar renal metabolic rewiring was observed in benign and malignant tumors derived from the impairment of mitochondrial complex 1 or GGT1 in human kidneys [17, 18]. In fact, metabolic rewiring causes more severely altered renal GSH metabolism in malignant tumors than in benign tumors. To that extent, we herein determined that the fold changes in the relative abundances of GSH and oxidized GSH in the older mice compared to younger mice were less than those previously reported in tumors compared to normal samples. In renal cell carcinoma, the proposed reason for increasing GSH levels is the counteracting damaging ROS to sustain the viability and growth of the malignancy [29]. Aging and oncogenesis share several pathophysiological mechanisms, including genomic instability, telomere shortening, proteostasis dysfunction, nutrient sensing and alterations of many cellular metabolism pathways [30]. The mechanisms of aging also occur in oncogenesis despite their divergent end results. Moreover, cancer is hypothesized to be relatively rare in young mammals due to the loss of tumor suppressor mechanisms [31]. Therefore, drawing the same conclusion from our mouse kidney aging study as that drawn from human kidney tumor models in previous studies is acceptable, and the concordant results are potentially due to their shared alteration of renal GSH metabolism.

In rat and mice, the hepatic gamma-glutamyl transferase (gamma-GT) activity is much lower than that in the kidneys [32, 33]; oxidation state of extracellular fluids depends on the catabolism of GSH by the renal γ-glutamyl transpeptidase (γ-GT) activity [34]. Gamma-GT is the enzyme present in the cell membranes that catalyzes the first step in GSH catabolism. Renal GSH concentrations are maintained by intracellular synthesis via the GSH cycle and transport of extracellular GSH [8]. The kidneys are known to be dependent on renal GSH levels to maintain restrained metabolism due to high rates of ROS production, specifically in the proximal tubules [8]. A significantly increased level of GSH has been recognized as a major metabolic determinant of renal cell carcinoma (RCC) [29]. In addition, since cysteine is a particularly unstable amino acid, GSH plays an important role in providing a cysteine reservoir [35]. Therefore, renal GSH catabolism is tightly associated with the interorgan metabolism of GSH to maintain systemic cysteine and GSH homeostasis. It is also well established that the concentration of systemic GSH decreases over time [36]. Collectively, we cautiously postulate a hypothesis that a significantly increased GSH pool in aged mouse kidneys is associated with an age-related systemic decline in circulating GSH levels. Notably, the recently published study hypothesized that age-dependent decline of low molecular weight (LMW) thiols, such as GSH, of the extracellular fluids could promote the protein-protein interaction of CoV-2 and the host cell, and increase the risk of infection of COVID-19 [34]. Furthermore, chronic kidney disease (CKD) is known to be associated with increased risk of severe COVID-19 infection [37]. Thus, the association between CKD and risk of severe COVID-19 infection could be related to the decrease in circulating GSH of the patients.

In addition to our findings in age-related metabolomic changes, we identified age-related differential gene expression by comparing the kidneys of mice of different ages. Genes residing in pathways governing immune system were specifically up-regulated in aged group. It is widely accepted that age is a major detrimental factor in the development of autoimmune disease [38]. The expression of autoantibodies also increases with age [39]. Furthermore, specifically in kidney, a previous study histologically identified an increase in the influx of glomerular macrophage from healthy aged mice [2]. Intriguingly, a recent study revealed that glutathione induces the stimulation of immune response by promoting macrophage polarization via acting as an ROS scavenger [40]. Defective antigen processing and presentation is known to be induced by the alteration of redox potential due to the changes in the level of intracellular GSH [41]. It is notable that we observed renal glutathione level increases and higher expression levels of genes in pathways related to the immune system in our aged mouse group. In fact, the expected redox potential calculated from the ratio of reduced GSH to oxidized GSH was decreased in aged mouse kidneys, similar to the results observed in renal tumor samples from previous human studies [17, 18]. Collectively, although whether rewired renal GSH metabolism is associated with increased immune signals in aged mice remains unclear, our results underscore the pivotal role of this metabolic rewiring and suggest that further research should be performed on the interplay between increased GSH concentrations and immune system stimulation in aged mouse kidneys.

We herein investigated rewired kidney metabolism attributed to aging by integrating metabolic profiling and transcriptomic analyses. We have shown that altered GSH metabolism is a major metabolic hallmark in aged kidneys that is associated with abnormal immune system stimulation. Also we hypothesized that the age related alteration in renal GSH metabolism may increase the potential risk of severe COVID-19 infection in aged individuals. Previous studies have demonstrated the role of the kidneys in GSH metabolism, the significance of GSH in the immune system, decreases in GSH levels over time, and the relationship between the immune system and the aging process. However, notably, our mouse kidney aging study is the first to demonstrate aging-related metabolic rewiring in mammalian kidney tissue and underscores the importance of rewired renal GSH metabolism in aging. This study does have several limitations, such as the utilization of a nonhuman system mimicking human aging and the absence of hypothesis validation by using controlled cell models. Since mimicking aged kidneys *in vitro* is not straightforward, aging-related renal metabolic rewiring was not further experimentally verified. Also, C57BL/6 mouse strain-specific character could affect our results. Moreover, obtaining healthy human kidney tissue samples over time for research purposes is technically challenging. Thus, the future establishment of an ex vivo kidney aging model (e.g., organoid culture) would feasibly help to further elucidate the mechanism underlying renal GSH metabolism alterations during the aging process.

## MATERIALS AND METHODS

### Mouse samples

All animal experiments were performed according to protocols approved by the Animal Care and Use Committee of the Korea Research Institute of Bioscience and Biotechnology (KRIBB). Young (3 months) and old (24 months) naive C57BL/6 male mice were obtained from the Laboratory Animal Resource Center of KRIBB. The mice were fed a standard diet and housed under a controlled temperature at 22−24°C and a 12 h light/dark cycle with humidity levels varying between 40 and 60% for 2 weeks. Both groups of mice were fasted for 12 hours before tissue sampling. Blood samples were collected at 10:00 am, and then kidney tissues were dissected and frozen in liquid nitrogen. Kidney tissues were stored at −80°C until processed. The body weights of the individual mice are provided in Supplementary Table 5. The mice increased their body weight over time similar to the bodyweight information for C57BL/6J mouse from the Jackson Laboratory [42, 43]. Also, we confirmed that renal glutathione intensity is not correlated with body weight, which is a proxy measure for obesity.

### LC-MS-based global profiling of kidney metabolites

The metabolites were extracted according to modified procedures previously used for mouse and rat tissues [44]. Briefly, whole kidney tissue was mixed with the extraction solvent at a ratio of 1:7.94 (ratio of kidney weight to water/methanol/chloroform (1/2.3/1.8, v/v/v)). Then, the mixture was homogenized twice with 2.8-mm zirconium oxide beads at 5,000 rpm for 25 s using a Precellys 24 tissue grinder (Bertin Technologies, France) and stored at 4°C for 20 min to separate the two phases. After centrifugation at 12,500 rpm and 4°C for 20 min, 87.5% of the supernatant was collected from each sample, equally aliquoted into 4 tubes and dried in a vacuum concentrator. One aliquot was reconstituted in 20% aqueous acetonitrile, and the solvent volume (μL) was 7.5-fold higher than the kidney weight (mg).

Chromatographic separation of kidney metabolites was carried out on an Acquity UPLC system (Waters, Milford, MA, USA) using an Acquity UPLC HSS T3 column (2.1 × 100 mm, 1.8 μm; Waters) at 40°C and a flow rate of 0.45 mL/min. The mobile phases comprised water containing 0.1% formic acid (solvent A) and acetonitrile containing 0.1% formic acid (solvent B). The UPLC gradient was programmed as follows: 1% to 10% B from 0 min to 3 min, 10% to 30% B from 3 min to 5 min, 30% to 50% B from 5 min to 10 min, 50% to 70% B from 10 min to 13 min, 70% to 90% B from 13 min to 15 min, 90% to 1% B from 15 min to 16 min, and 1% B for 2 min to equilibrate for the next run. A total of 5 μL of the sample was injected using the partial loop mode for both positive and negative ionization modes. Quality control (QC) samples, which were pooled identical sample aliquots, were measured regularly throughout the experiment for data reproducibility.

A triple TOF™ 5600 MS/MS system (Sciex, Concord, Canada) equipped with a DuoSpray ion source operating in the positive and negative electrospray ionization (ESI) modes was used for the detection of kidney metabolites with a mass range of *m/z* 50–1,000. The following parameter settings were used: ion spray voltage, 5,500 V (positive mode) and 4,500 V (negative mode); source temperature, 500°C; nebulizer gas pressure, 50 psi; drying gas pressure, 60 psi; curtain gas pressure, 30 psi; declustering potential, 90 eV; and accumulation time, 100 ms. An automated calibrant delivery system (Sciex) was used to maintain mass accuracy with an atmospheric pressure chemical ionization calibration solvent (Sciex). MS/MS spectra for ions were obtained via the information-dependent acquisition (IDA) method.

The MS spectral data were processed by MarkerView™ (Sciex, Concord, Canada) to identify peaks, perform peak alignment, and generate peak tables of the m/z values and retention times (min). The data were normalized using the total spectral area. After excluding isotopes, peaks with coefficients of variation below 20 in the QC samples were selected to identify reliable peaks and prohibit instrumental bias.

For the identification of metabolites, fragment patterns (MS/MS spectra) were initially matched to the *in silico* fragments generated from MS-DIAL and MS-Finder. Then, metabolites were tentatively identified by comparing the experimental data to the METLIN database (https://metlin.scripps.edu).

### Serum glutathione quantification

To extract metabolites in serum, 20 μL of the serum sample was mixed with 220 μL of chloroform/methanol (2:1, v/v) and 40 μL of water. The mixture was vortexed and incubated at 4°C for 10 min. After centrifugation, 40 μL of the upper aqueous supernatant was dried under a nitrogen concentrator. The extracts were diluted with 80 μL of a methanol/water mixture (20:80, v/v) for targeted analysis. To quantify the metabolites, liquid chromatography-mass spectrometry was performed on an Agilent 1290 Infinity LC and an Agilent 6490 Triple Quadrupole MS system equipped with an Agilent Jet Stream ESI source (Agilent Technologies, USA). MassHunter Workstation (Ver B.07.01 Agilent Technologies, USA) software was used for data acquisition and analysis. LC separations were carried out on an Imtakt SM C18 column (100 × 2.0 mm, particle size 3.0 μm, Imtakt, USA), with the column temperature and flow rate set to 25°C and 0.2 mL/min, respectively. The binary gradient system comprised 0.1% formic acid in water (solvent A) and 0.1% formic acid in methanol (solvent B). The linear gradient program was as follows: 5% B from 0–3 min, 5–100% B from 3–14 min, 100% B from 14–16 min, 100–5% B from 16–17 min, and 5% B from 17–20 min. The injection volume of the sample was 1 μL. Tandem MS experiments were conducted in positive ion mode with the following parameters: capillary voltage, 3.5 kV; nebulizer gas, nitrogen at 40 psi; drying gas temperature, 120°C; drying gas flow rate, 11 L/min; sheath gas temperature, 350°C; sheath gas flow rate, 12 L/min; and nozzle voltage, 500 V. The selected reaction monitoring (SRM) of glutathione was a m/z 308 > 179.1 transition at a collision energy of 10eV.

### Transcriptome sequencing

The transcriptomes of mouse kidney tissue samples were analyzed by Macrogen. Triplicate kidney tissue samples from young and old mice were stored on dry ice before sequencing. The mm10 data set was used as the sequencing reference genome, and annotation was performed based on NCBI-108 data. The TruSeq Stranded mRNA LT Sample Prep Kit and TrueSeq Stranded mRNA Sample Preparation Guide, Part # 15031047 Rev. E were used as the library kit and library protocol, respectively. Reagents were obtained from the NovaSeq 600 S4 Reagent Kit, and the sequencing protocol was obtained from NovaSeq 6000 System User Guide Document # 1000000019358 v02. After the QC processing of sequencing data, trimmed reads were mapped to the reference genome by using HISAT2, and StringTie was used for transcript assembly.

### Network analysis

Metabolomics data were processed to build a correlation coefficient-based network. Identified metabolites are represented as nodes in the network graph. The resampling strategy-based probabilistic context likelihood of relatedness (PCLR) algorithm [13] and R software [45] were applied to determine the edges between nodes. The built network was further processed by using Cytoscape [46]. The width and height of the nodes were scaled based on the stress centrality measures, where stress centrality was the number of shortest paths passing through as a proxy measurement to capture the importance of features [14]. Differential network analysis was also performed to evaluate differences in networks between older mice and younger mice according to the study of Sanjeevan et al. [47].

### Pathway analysis

Pathway analysis of the metabolomics results was conducted through MetaboAnalyst [48]. The relative intensities of metabolites with Human Metabolome Data Base (HMDB) identifiers were used as input for the analysis. A hypergeometric test was conducted to evaluate the pathway enrichments based on *Mus musculus* data in the Kyoto Encyclopedia of Genes and Genomes (KEGG) database [49]. Pathway analysis of the metabolomics results was conducted using the gage package in R software [50].

### Gene set clustering analysis

We performed gene set clustering analysis by using GScluster to identify the gene set-gene set interactions [21]. Adjusted *p* values from the Deseq2 results, the KEGG gene set database for *Mus musculus* and the protein-protein interaction (PPI) network for *Mus musculus* from STRING were used for the clustering analysis [51]. The GsQCutoff and GQCutoff were set to 0.9.

### Gene set enrichment analysis

Gene set enrichment analysis (GSEA) was conducted by using GSEA 4.1.0 software [22]. Gene sets were downloaded from MSigDB [23].

### Statistical analysis

Principal component analysis (PCA) and partial least squares discriminant analysis (PLS-DA) were carried out using SIMCA-P+ software version 12.0 (Umetrics, Umeå, Sweden) to visualize score plots and identify differences between the young and old groups. The Wilcoxon rank sum test or *t*-test was used to evaluate significant differences in metabolite or RNA expression levels between the young and old groups. Intragroup metabolite level differences in a pathway were evaluated by using 2-way ANOVA with Sidak’s multiple comparisons test implemented in GraphPad. R 1.2.1335 software and relevant packages were utilized for the statistical analyses and figure generation in this research [45]. Gene ontology analysis was conducted by using the goseq R package [52].

## Supplementary Materials

Supplementary Figures

Supplementary Tables
