# Supplementary material for: Glutathione is an aging-related metabolic signature in the mouse kidney
Source: Aging (Albany NY). 2021 Sep 7;13(17):21009–28. doi: 10.18632/aging.203509 (PMC8457589; doi:10.18632/aging.203509)
Supplement: Supplementary Figures [file aging-13-203509-s001.pdf]

SUPPLEMENTARY FIGURES

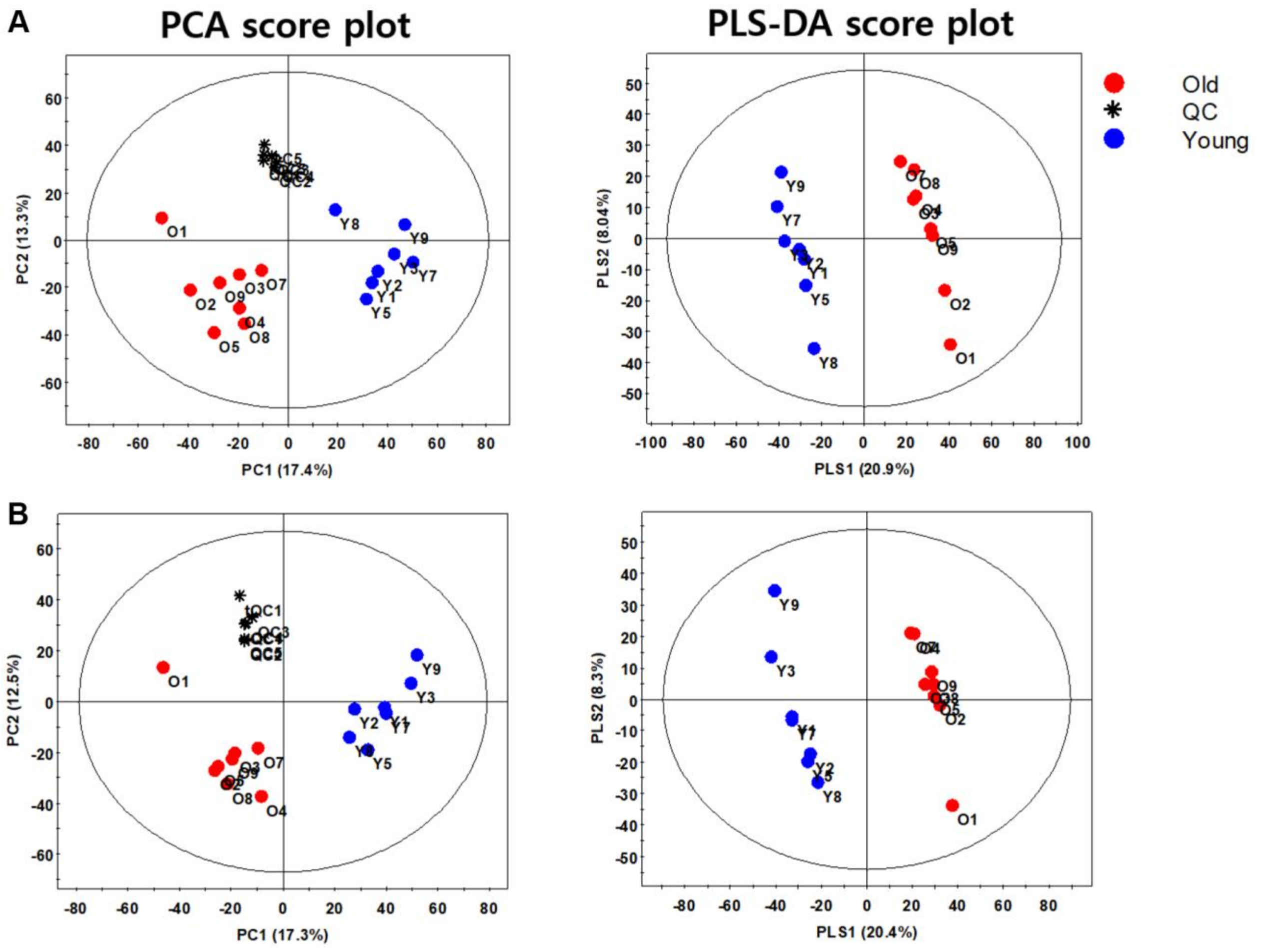

Supplementary Figure 1. Score plots of principal component analysis (PCA) and partial least squares discriminant analysis (PLS-DA) obtained from the UPLC-QTOF MS spectra of kidney extracts. (**A**) PCA (*R2X* = 47.3%, *Q2* = 7.7%) and PLS-DA (*R2X* = 29.0%, *R2Y* = 99.4%, *Q2* = 84.2%) score plots for the positive ion mode. (**B**) PCA (*R2X* = 38.3%, *Q2* = 6.5%) and PLS-DA (*R2X* = 28.7%, *R2Y* = 99.4%, *Q2* = 86.2%) for the negative ion mode.

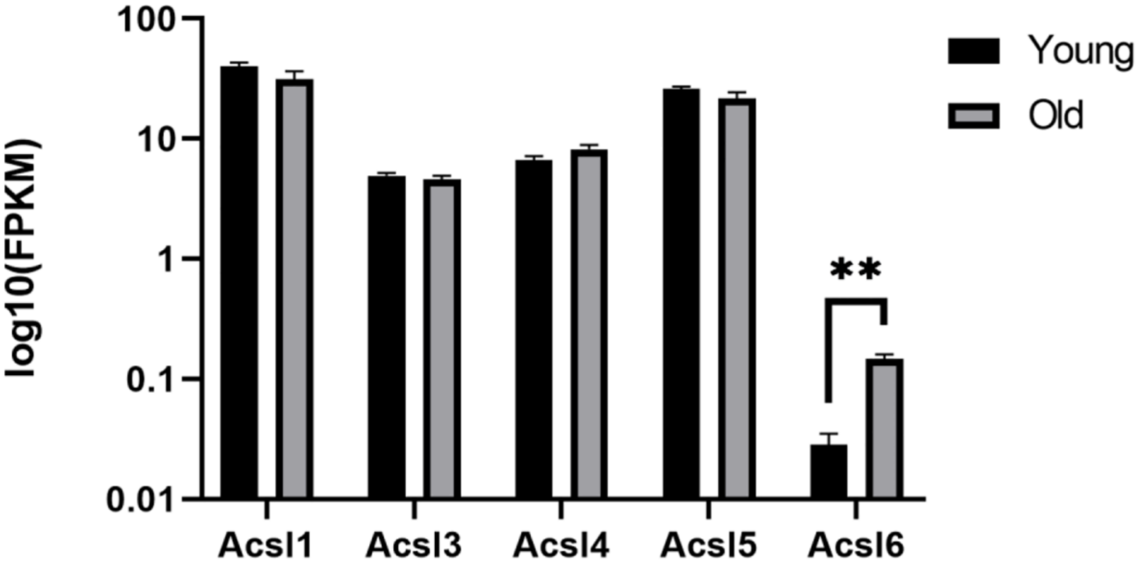

Supplementary Figure 2. RNA-seq results of long-chain-fatty-acid-CoA ligase (Acsl) genes. FPKM values of genes from the old (*n* = 3) and young (*n* = 3) groups were shown. The mean ± SEM of each group is shown. Adjusted *p*-values were calculated by using the Deseq2 R package. (*p*-value: *< 0.05, **< 0.01, ***< 0.001, ****< 0.0001).

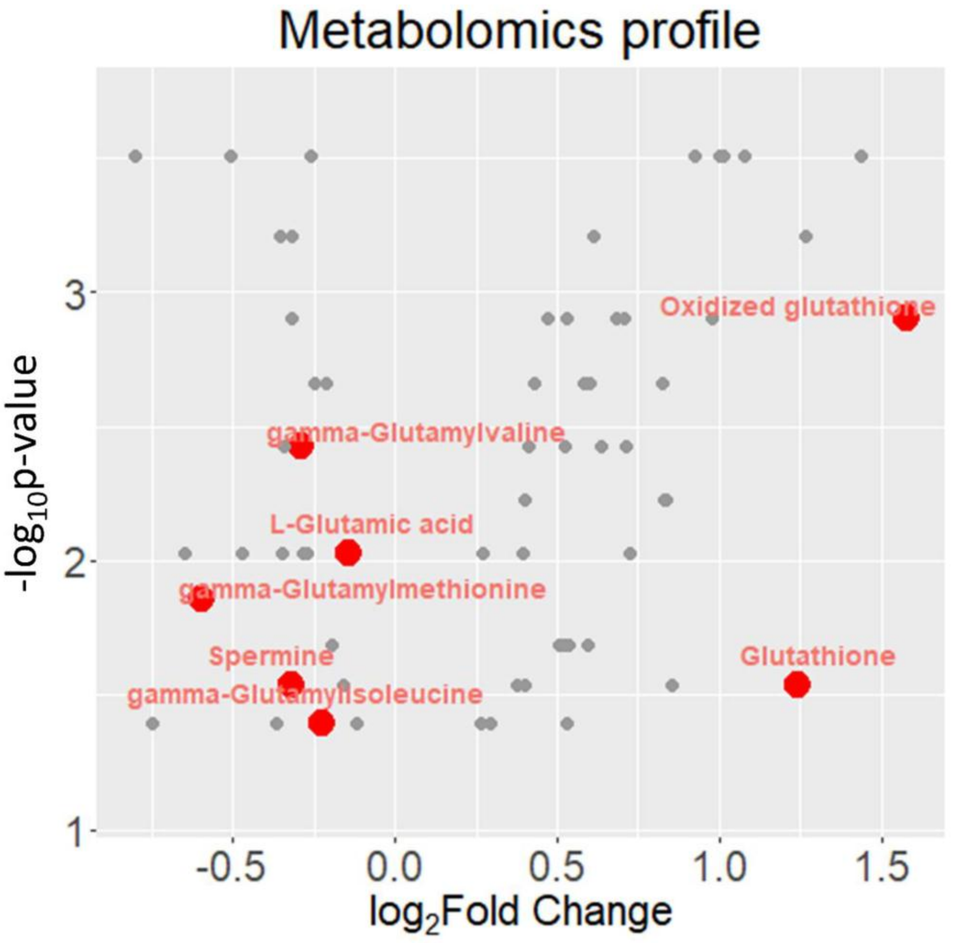

Supplementary Figure 3. Volcano plot of the LC-MS-based aqueous metabolite profiling results. Glutathione, oxidized glutathione and gamma-glutamyl amino acids are denoted in red.

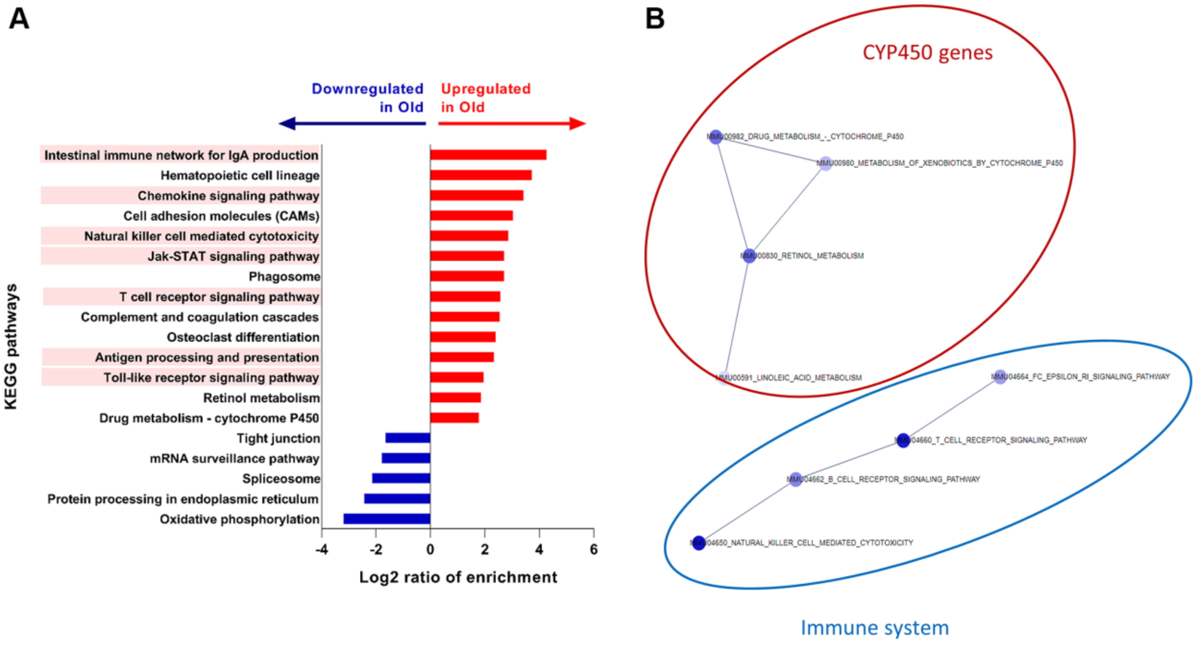

Supplementary Figure 4. Transcriptomic analysis results. Pathway enrichment analysis results using the KEGG database (**A**). Upregulated metabolic pathways in the old group are plotted as red bars, and downregulated metabolic pathways in the old group are plotted as blue bars. Pathway-pathway interaction analysis results using the clustering-based method (**B**). CYP gene-related pathways were grouped and colored red, and immune system-related pathways were grouped and colored blue.

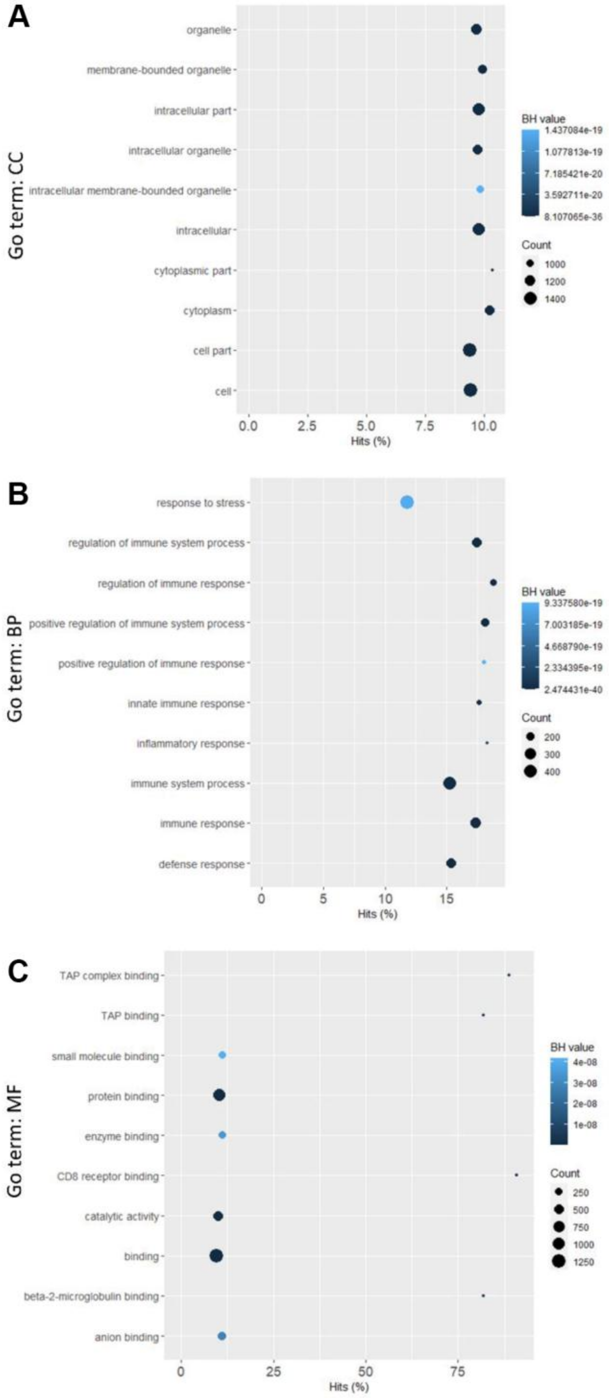

Supplementary Figure 5. Gene Ontology analysis results for the cellular component (**A**), biological process (**B**) and molecular function (**C**) categories. The FPKM values of genes from the old (*n* = 3) and young (*n* = 3) groups were used for the analysis.
