# Supplementary material for: Glutathione is an aging-related metabolic signature in the mouse kidney
Source: Aging (Albany NY). 2021 Sep 7;13(17):21009–28. doi: 10.18632/aging.203509 (PMC8457589; doi:10.18632/aging.203509)
Supplement: Supplementary Tables [file aging-13-203509-s002.pdf]

SUPPLEMENTARY TABLES

Supplementary Table 1. LC-MS-based metabolomic analysis results of metabolic genes related to glutathione metabolism in mouse kidney tissue.

rt	mz	Metabolite	Wilcox_p	*t*-test_p	FC
1.53	307.0843	Oxidized glutathione	0.01	0.00	2.77
1.12	308.0915	Glutathione	0.03	0.03	2.37
0.61	148.0603	L-Glutamic acid	0.01	0.01	0.91
3.71	261.1447	ϒ-glutamylisoleucine	0.04	0.04	0.86
2.26	247.1285	ϒ-glutamylvaline	0.01	0.00	0.82
2.3	279.1007	ϒ-glutamylmethionine	0.02	0.01	0.66
1.29	497.1009	Bis-ϒ-glutamylcystine	0.01	0.01	0.64

The retention times (rts) and detected mass over charge (mz) values of metabolites are shown. Fold changes (FCs) were calculated by dividing the mean relative intensity values of identified metabolites from the old group (n = 8) by those from the young group (n = 7). *P*-values were determined by the Wilcoxon rank sum test (Wilcox_p) and *t*-test (*t*-test_p) using R.

Supplementary Table 2. Metaboanalyst results.

	Total Cmpd	Hits	Raw p	FDR	Impact
Riboflavin metabolism	4	2	0.00078	0.00397	0.5
Nicotinate and nicotinamide metabolism	15	2	0.00004	0.00095	0.4290
Glutathione metabolism	28	4	0.00043	0.00281	0.3026
Purine metabolism	65	6	0.00037	0.00281	0.2061
Alanine, aspartate and glutamate metabolism	28	2	0.00820	0.01066	0.1971
Citrate cycle (TCA cycle)	20	2	0.00337	0.00572	0.1345
Arginine and proline metabolism	38	2	0.00140	0.00438	0.0860
Glyoxylate and dicarboxylate metabolism	32	3	0.00204	0.00482	0.0318
Pyrimidine metabolism	39	2	0.00092	0.00397	0.0202

The number of total annotated compounds in each metabolic pathway (Total Cmpd) and the number of identified metabolites (Hits) were used for the hypergeometrical test-based pathway enrichment test. According to the test results, raw *p*-values (Raw p) and FDR values were calculated. Additionally, the estimated impact parameters in the Metaboanalyst results are shown.

Supplementary Table 3. Transcriptomic results of metabolic genes related to glutathione metabolism in mouse kidney tissue.

Gene_Sym	Description	FC	Adjusted *p*-value
Ggct	gamma-glutamyl cyclotransferase	0.55	1.49E-06
Oplah	5-oxoprolinase (ATP-hydrolysing)	1.38	0.0406977
Gss	glutathione synthetase	0.8	0.0608817
Chac2	ChaC, cation transport regulator 2	0.78	0.0956953
Ggt1	gamma-glutamyltransferase 1	0.87	0.3969397
Anpep	alanyl (membrane) aminopeptidase	0.79	0.1122825
Gclc	glutamate-cysteine ligase, catalytic subunit	0.74	0.4305143
Dpep1	dipeptidase 1	0.75	0.4100684

Fold changes (FCs) were calculated by dividing the mean FPKM values of genes from the old group (n = 3) by those from the young group (n = 3). Adjusted *p*-values were calculated by using the Deseq2 R package.

Supplementary Table 4. Gene Set Enrichment Analysis results of transcriptome data from mouse kidney tissue.

Gene Set	ES	NES	NOM *p*-val	FDR *q*-val
GSE17721_LPS_VS_POLYIC_1H_BMDC_DN	–0.96	–1.53	0	0.046
GSE14308_TH1_VS_NAIVE_CD4_TCELL_DN	–0.9	–1.46	0	0.046
GSE43955_10H_VS_30H_ACT_CD4_TCELL_DN	–0.96	–1.42	0	0.046
GSE14308_INDUCED_VS_NATURAL_TREG_UP	–0.98	–1.36	0	0.069
GSE14769_UNSTIM_VS_120MIN_LPS_BMDM_DN	–0.98	–1.36	0	0.055
CAIRO_LIVER_DEVELOPMENT_DN	–0.91	–1.35	0	0.054
OHGUCHI_LIVER_HNF4A_TARGETS_DN	–0.96	–1.34	0	0.078
GSE19198_6H_VS_24H_IL21_TREATED_TCELL_DN	–0.93	–1.33	0	0.074

Nominal *p*-values (NOM *p*-val) were adjusted as FDR *q*-values. Only the gene sets with FDR *q*-values less than 0.1 are shown above. Enrichment scores (ES) and negative enrichment scores (NES) are also shown.

Supplementary Table 5. Body weights of the young and old mice.

Young (3 months)	BW (g)
Y1	25.2
Y2	25.8
Y3	28.4
Y5	21.6
Y7	23.5
Y8	22.4
Y9	24.3
**Old (24 months)**	
O1	37.7
O2	45.3
O3	35.6
O4	49.6
O5	31.0
O7	35.6
O8	45.1
O9	41.7

Young group (n = 7) and old group (n = 8).
